# Supplementary material for: Machine Learning for Optical Motion Capture-Driven Musculoskeletal Modelling from Inertial Motion Capture Data
Source: Bioengineering (Basel). 2023 Apr 24;10(5):510. doi: 10.3390/bioengineering10050510 (PMC10215337; doi:10.3390/bioengineering10050510)
Supplement: Supplementary file 1 [file bioengineering-10-00510-s001.zip › bioengineering-2321340-supplementary.pdf]

Supplementary Information: Machine Learning for Optical Motion Capture-driven Musculoskeletal Modelling from Inertial Motion Capture Data

Abhishek Dasgupta0000-0003-4420-0656, Rahul Sharma0000-0003-0700-3098, Challenger Mishra*0000-0001-
and Vikranth H. Nagaraja*0000-0001-7491-8242

April 24, 2023

2. Materials and methods

2.1 Data

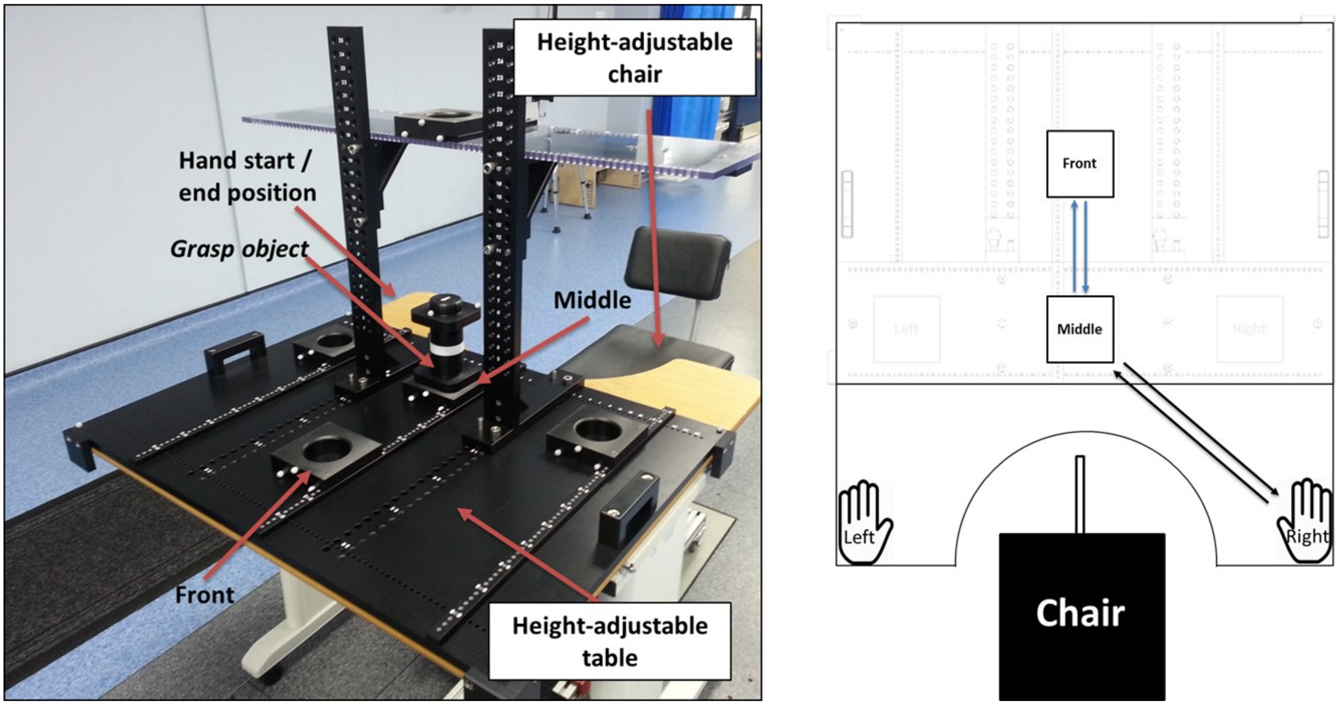

Figure S1: Left side: Custom-built apparatus for *Reach-to-Grasp* task execution in the Forward direction; Right-side: Reach-to-Grasp task setup.

3. Results

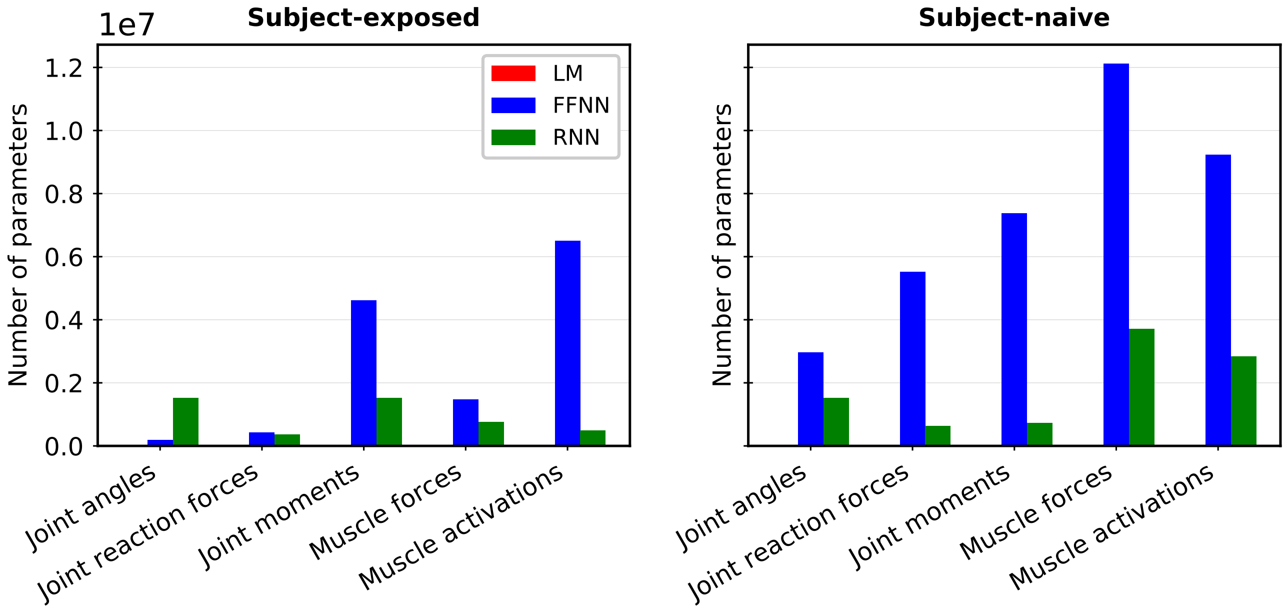

Figure S2: Number of parameters in Linear Model (LM), Feed-Forward Neural Network (FFNN), and Recurrent Neural Network (RNN). Note: The number of parameters for Linear Models is negligible than the other two models; hence, they are indiscernible here.

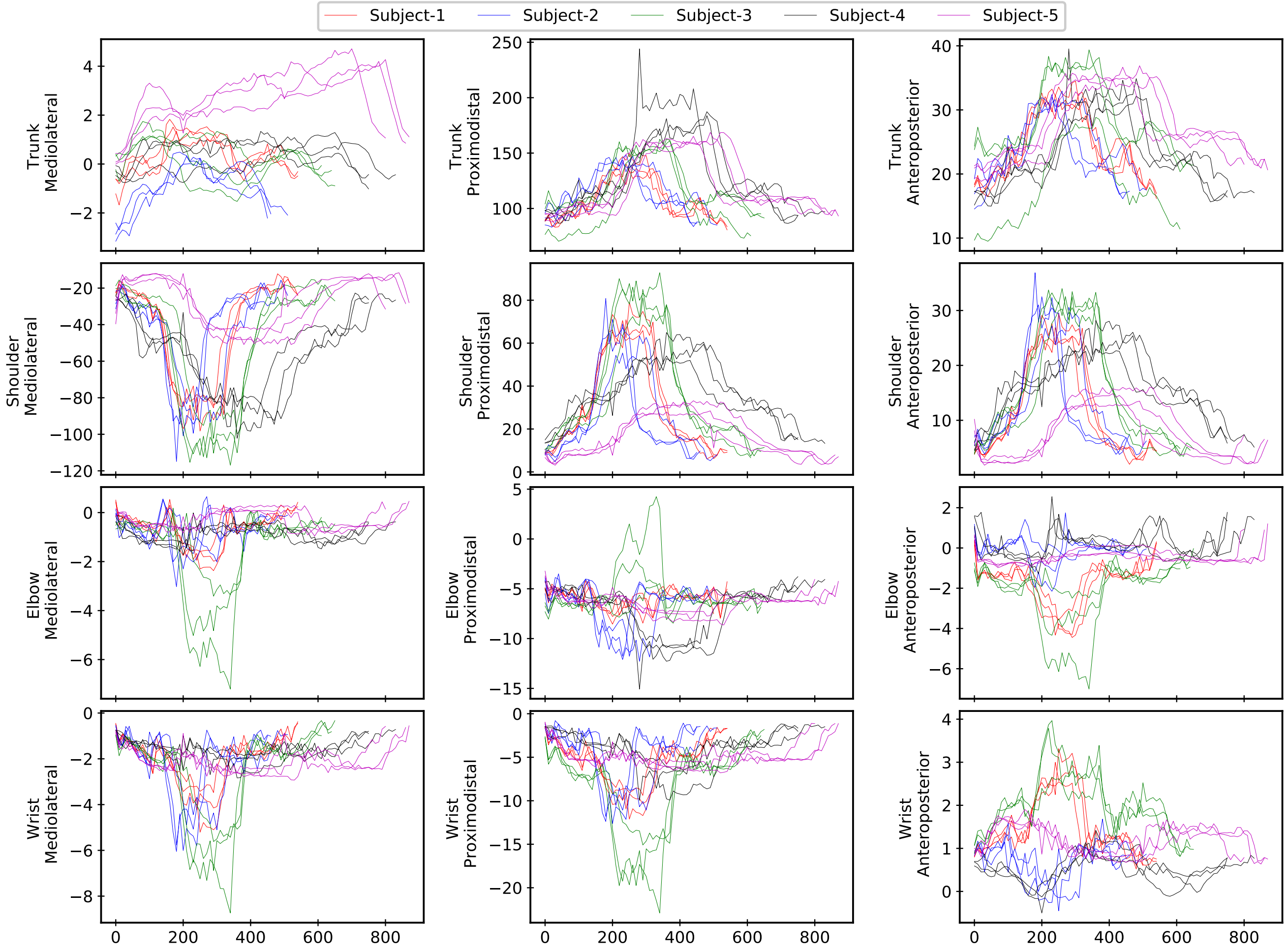

Figure S3: Joint reaction forces estimated by Musculoskeletal (MSK) model for all five subjects. Note: The three trials for individual subjects are shown in the same colour.

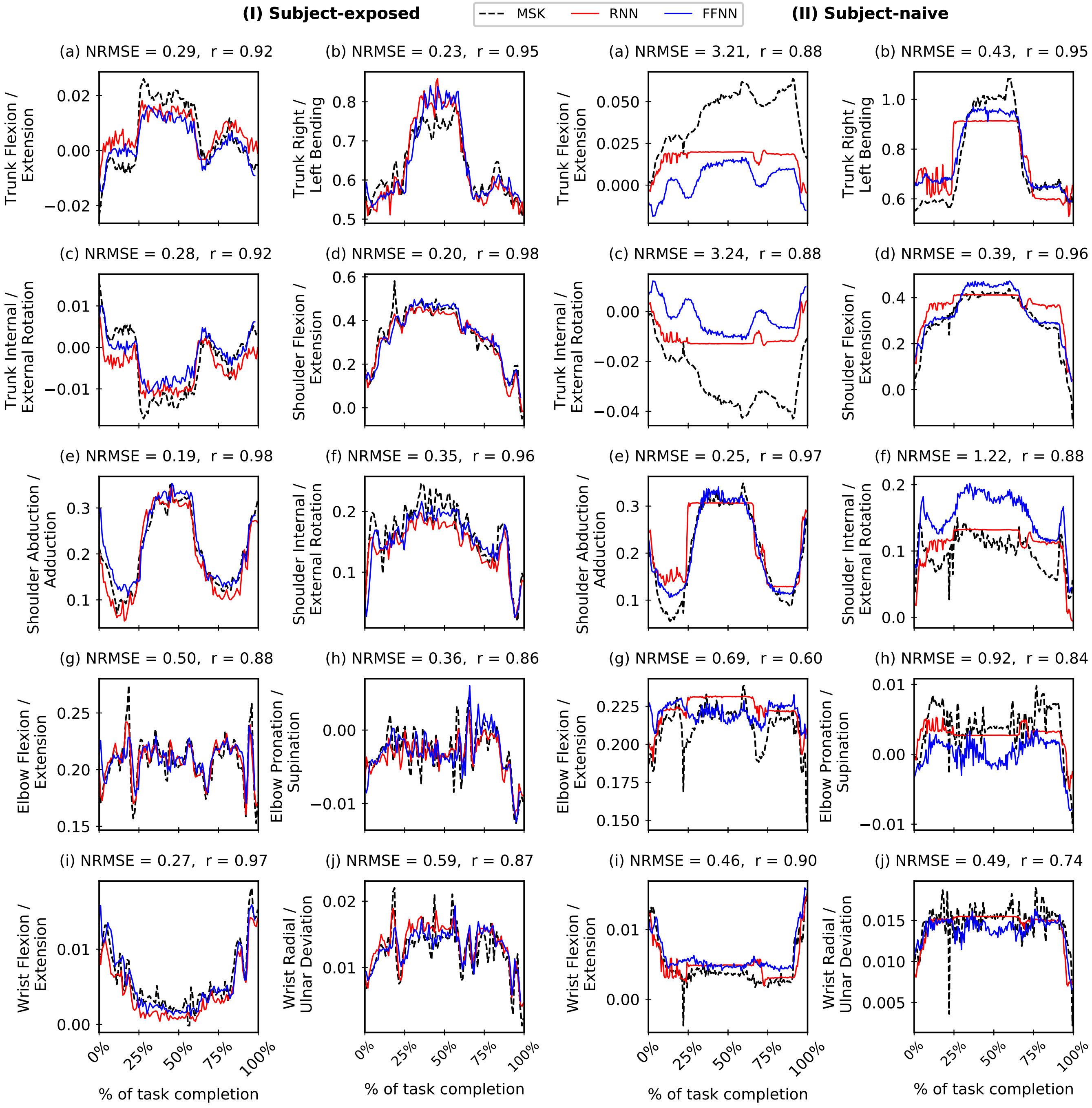

Figure S4: Comparing Feed-Forward Neural Network (FFNN) and Recurrent Neural Network (RNN) predictions for joint moments (% Body Weight × Body Height) with the corresponding Musculoskeletal (MSK) model outputs on a test trial in *Subject-exposed* (left) and *Subject-naive* (right) settings. Note: The performance of FFNN and RNN are comparable (see Figure 4 in the main text); in this figure, we report r and NRMSE values only for FFNN.

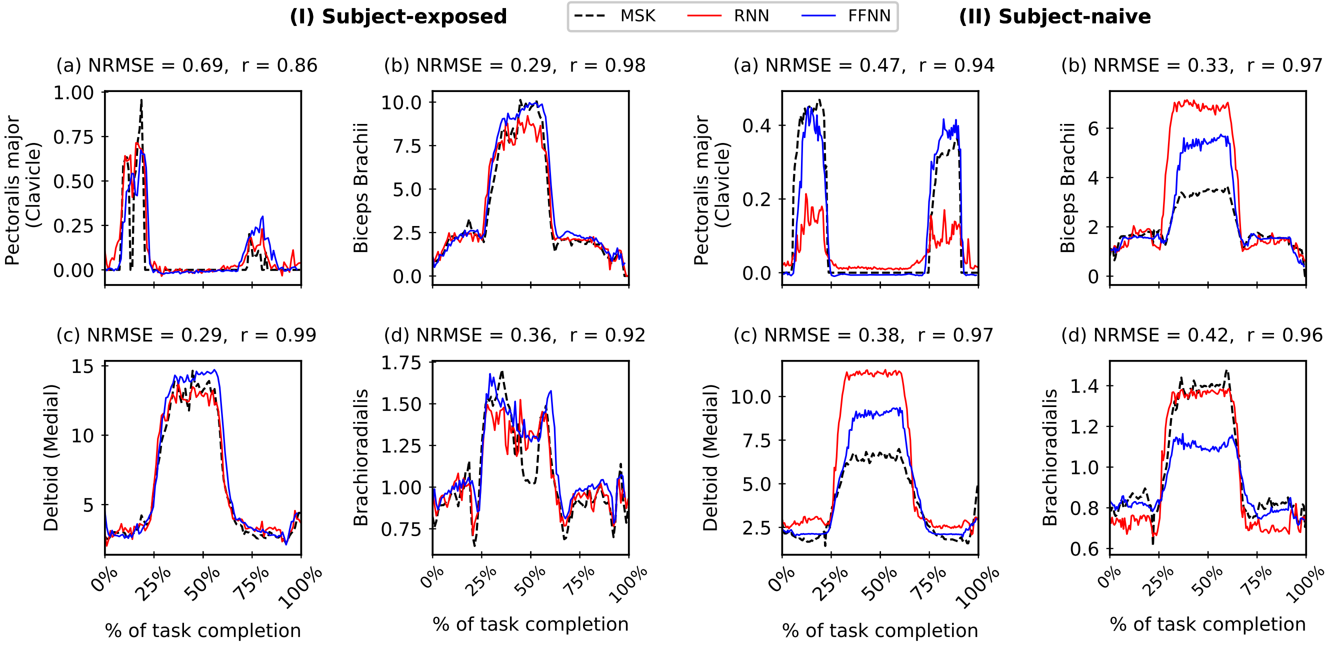

Figure S5: Comparing Feed-Forward Neural Network (FFNN) and Recurrent Neural Network (RNN) predictions for muscle forces (% Body Weight) with the corresponding Musculoskeletal (MSK) model outputs for a test trial in *Subject-exposed* (left) and *Subject-naive* (right) settings. Note: The performance of FFNN and RNN are comparable (see Figure 4 in the main text); in this figure, we report r and NRMSE values only for FFNN.

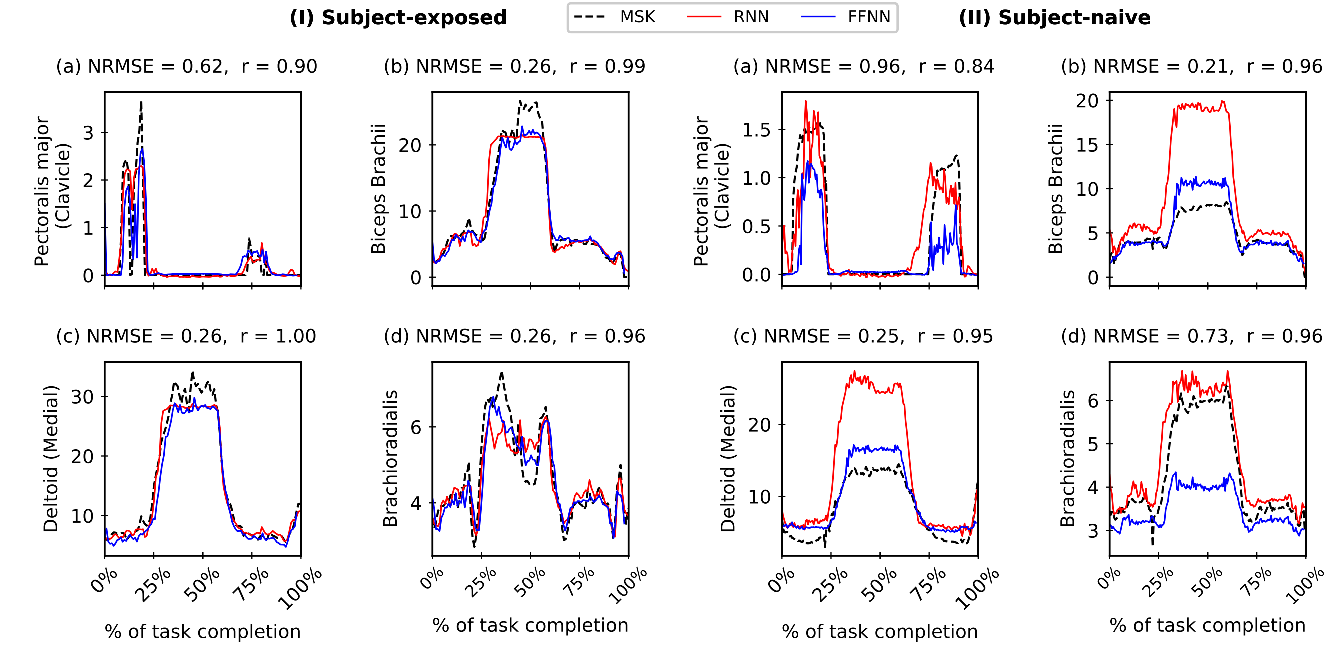

Figure S6: Comparing Feed-Forward Neural Network (FFNN) and Recurrent Neural Network (RNN) predictions for muscle activations (%) with the corresponding Musculoskeletal (MSK) model outputs for a test trial in *Subject-exposed* (left) and *Subject-naive* (right) settings. Note: The performance of FFNN and RNN are comparable (see Figure 4 in the main text); in this figure, we report r and NRMSE values only for FFNN.

	Linear Model		Feed-Forward Neural Network		Recurrent Neural Network	
Output	r_{avg} Mean (SD)	$NRMSE_{avg}$ Mean (SD)	r_{avg} Mean (SD)	$NRMSE_{avg}$ Mean (SD)	r_{avg} Mean (SD)	$NRMSE_{avg}$ Mean (SD)
	**Subject-exposed**					
Joint angles	0.85 (0.15)	0.64 (0.62)	0.92 (0.23)	0.25 (0.2)	0.93 (0.13)	0.32 (0.24)
Joint reaction forces	0.77 (0.21)	0.70 (0.31)	0.91 (0.12)	0.34 (0.11)	0.91 (0.11)	0.33 (0.09)
Joint moments	0.81 (0.11)	0.58 (0.25)	0.88 (0.08)	0.41 (0.2)	0.87 (0.09)	0.46 (0.2)
Muscle forces	0.73 (0.31)	0.64 (0.27)	0.85 (0.29)	0.37 (0.17)	0.83 (0.3)	0.33 (0.17)
Muscle activations	0.75 (0.28)	0.74 (0.28)	0.88 (0.25)	0.29 (0.15)	0.84 (0.28)	0.37 (0.17)
	**Subject-naive**					
Joint angles	0.85 (0.11)	2.54 (2.22)	0.86 (0.17)	0.82 (0.65)	0.86 (0.13)	1.10 (1.02)
Joint reaction forces	0.5 (0.59)	1.34 (0.98)	0.75 (0.35)	0.90 (0.74)	0.64 (0.34)	0.84 (0.77)
Joint moments	0.84 (0.11)	1.57 (0.68)	0.85 (0.12)	1.12 (1.02)	0.75 (0.13)	0.89 (0.68)
Muscle forces	0.71 (0.2)	1.70 (1.20)	0.95 (0.03)	0.49 (0.53)	0.93 (0.04)	0.58 (0.27)
Muscle activations	0.70 (0.17)	1.78 (1.16)	0.93 (0.04)	0.42 (0.28)	0.92 (0.06)	0.61 (0.21)

Table S1: Average Pearson's correlation coefficient and average NRMSE values for Linear Model (LM), Feed-Forward Neural Network (FFNN), and Recurrent Neural Network (RNN) prediction compared with Musculoskeletal (MSK) model outputs. The FFNN and RNN models consistently outperform the linear model. For a given output category, averaging is done over all output features and test trials.

		r					NRMSE				
Output	ML model	Mean	SD	Max	Min	IQR	Mean	SD	Max	Min	IQR
		**Subject-exposed**									
Joint angles	FFNN	0.92	0.23	1.0	-0.07	0.04	0.25	0.2	0.79	0.04	0.28
	B-LSTM	0.93	0.13	0.99	0.38	0.07	0.32	0.24	0.76	0.03	0.45
Joint reaction forces	FFNN	0.91	0.12	0.99	0.46	0.07	0.34	0.11	0.55	0.17	0.13
	LSTM	0.91	0.11	0.99	0.51	0.09	0.33	0.09	0.49	0.18	0.16
Joint moments	FFNN	0.88	0.08	0.98	0.7	0.1	0.41	0.2	0.97	0.19	0.16
	B-LSTM	0.87	0.09	0.98	0.66	0.14	0.46	0.20	1.06	0.22	0.15
Muscle forces	FFNN	0.85	0.29	0.99	0.07	0.08	0.37	0.17	0.69	0.10	0.18
	LSTM	0.83	0.30	0.99	0.05	0.09	0.33	0.17	0.74	0.18	0.09
Muscle activations	FFNN	0.88	0.25	1.0	0.21	0.05	0.29	0.15	0.62	0.08	0.07
	LSTM	0.84	0.28	0.99	0.11	0.09	0.37	0.17	0.71	0.08	0.08
		**Subject-naive**									
Joint angles	FFNN	0.86	0.17	0.98	0.32	0.07	0.82	0.65	2.42	0.20	0.60
	B-LSTM	0.86	0.13	0.98	0.56	0.09	1.10	1.02	3.56	0.20	0.73
Joint reaction forces	FFNN	0.75	0.35	0.98	-0.61	0.24	0.9	0.74	3.24	0.2	0.51
	LSTM	0.64	0.34	0.92	-0.24	0.31	0.84	0.77	3.19	0.15	0.51
Joint moments	FFNN	0.85	0.12	0.99	0.56	0.18	1.12	1.02	3.24	0.25	0.72
	GRU	0.75	0.13	0.94	0.5	0.20	0.89	0.68	2.50	0.35	0.22
Muscle forces	FFNN	0.95	0.03	0.97	0.88	0.02	0.49	0.53	1.86	0.09	0.23
	GRU	0.93	0.04	0.97	0.85	0.07	0.58	0.27	1.12	0.24	0.31
Muscle activations	FFNN	0.93	0.04	0.96	0.84	0.04	0.42	0.28	0.96	0.10	0.30
	LSTM	0.92	0.06	0.97	0.78	0.07	0.61	0.21	0.87	0.31	0.33

Table S2: Average Pearson's correlation coefficient and Average NRMSE Values for Feed-Forward Neural Network (FFNN) and Recurrent Neural Network (RNN) predictions compared with Musculoskeletal (MSK) model outputs. Note: The average is taken over all output features and over all test trials (for a given output category). In the RNN cell category, 'B' stands for Bidirectional cell (which processes the input time series in both forward and backward directions).

Output	ML model	RMSE				
		Mean	SD	Max	Min	IQR
		**Subject-exposed**				
Joint angles (degrees)	FFNN	2.91	1.86	8.24	1.07	1.49
	B-LSTM	3.42	1.73	7.91	1.04	2.32
Joint reaction forces (% Body Weight)	FFNN	2.15	2.55	9.75	0.21	2.37
	LSTM	2.08	2.37	7.84	0.24	2.38
Joint moments (% Body Weight × Body Height)	FFNN	0.02	0.02	0.06	0.00	0.02
	B-LSTM	0.02	0.02	0.07	0.00	0.03
Muscle forces (% Body Weight)	FFNN	0.64	0.6	1.53	0.01	1.01
	LSTM	0.45	0.37	0.97	0.03	0.64
Muscle activations (%)	FFNN	0.97	0.68	1.92	0.04	1.19
	LSTM	1.38	1.05	2.78	0.04	1.85
		**Subject-naive**				
Joint angles (degrees)	FFNN	9.07	3.54	16.49	4.31	5.21
	B-LSTM	11.09	4.62	20.37	3.40	6.07
Joint reaction forces (% Body Weight)	FFNN	5.52	6.74	26.57	0.28	6.00
	LSTM	6.44	10.81	41.45	0.35	6.34
Joint moments (% Body Weight × Body Height)	FFNN	0.03	0.03	0.09	0.00	0.05
	GRU	0.03	0.03	0.11	0.00	0.03
Muscle forces (% Body Weight)	FFNN	0.48	0.46	1.38	0.06	0.54
	GRU	1.13	1.09	2.90	0.08	2.06
Muscle activations (%)	FFNN	1.09	0.65	2.03	0.17	1.02
	LSTM	3.03	2.82	7.26	0.28	5.17

Table S3: Average RMSE Values for Feed-Forward Neural Network (FFNN) and Recurrent Neural Network (RNN) predictions compared with Musculoskeletal (MSK) model outputs. Note: The average is taken over all output features and over all test trials (for a given output category). In the RNN cell category, 'B' stands for Bidirectional cell (which processes the input time series in both forward and backward directions).
